# Vitamin A and D Status, Selected Genetic Variants, and Allergy-Related Phenotypes: An Exploratory Retrospective Study

**DOI:** 10.3390/diagnostics16203277

**Published:** 2026-10-09

**Authors:** Gulsen Meral, Esma Gokcen Alper Acar, Neval Burkay, Bilge Ozluer Baser, Ahmet Avcı, Ruya Atesli

**Affiliations:** 1 Epigenetic Coaching, London SW10 9EL, UK; 2 Department of Pediatrics, Faculty of Medicine, Istanbul Nisantasi University, Istanbul 34398, Turkey; 3 Department of Statistics, Mimar Sinan Fine Arts University, Istanbul 34349, Turkey; 4 Department of Biostatistics, Cerrahpaşa Faculty of Medicine, Istanbul University, Istanbul 34098, Turkey; 5 Department of Pediatrics, Acibadem Kent Hospital, Izmir 35630, Turkey

**Keywords:** allergy-related phenotypes, vitamin D, vitamin A, genetic polymorphisms, *VDR*, *GC*, *CYP2R1*, *BCMO1*, personalized nutrition

## Abstract

**Background/Objectives:** Vitamins A and D are critical regulators of immune function, and genetic variation in their metabolic pathways may influence vitamin status and immune responses. This study investigated the combined nutrigenetic roles of vitamins A and D and their association with allergy-related phenotypes. **Methods:** In this retrospective study, 80 individuals were included (40 with allergy-related phenotypes and 40 controls). Genetic polymorphisms in *CYP2R1*, *GC*, *VDR*, and *BCMO1* were analyzed. Serum vitamin A and 25(OH)D concentrations were compared between groups, and multivariable analyses were performed to assess the effects of age and sex. Genotype distributions were evaluated using chi-square tests, and co-occurring genetic patterns were explored using the Apriori algorithm (*p* < 0.05). **Results:** Both serum vitamin A and 25(OH)D concentrations were significantly lower in the allergy-related phenotypes. Genotype differences were observed for *CYP2R1* rs2060793 and *BCMO1* rs12934922; however, only *CYP2R1* rs2060793 remained significant after FDR correction. Although residual confounding cannot be ruled out, exploratory association rule mining identified co-occurrence patterns involving *GC*, *VDR*, and *CYP2R1* variants with vitamin D deficiency and *BCMO1* variants with vitamin A deficiency. These findings should be considered preliminary and hypothesis-generating rather than evidence of established or causal genetic effects. **Conclusions:** These preliminary findings indicate potential associations between vitamin status, specific genetic variants, and allergy-related phenotypes. Because of the study’s retrospective design and limited sample size, these genetic observations should be regarded as hypothesis-generating and warrant validation in larger, prospectively characterized cohorts.

## 1. Introduction

Allergic diseases burden healthcare globally [1]. Factors such as vitamin/mineral deficiencies, environmental exposures, diet, and the gut microbiota influence their onset [2]. Vitamins A and D, vital for immunity, are attracting attention. Genetic variants in their metabolism may affect immune disorders, including allergies [3,4,5,6]. These findings underscore the importance of nutriepigenetics in personalized medicine.

Vitamin D is vital for bone and calcium homeostasis and for biological functions such as cell growth, vascular health, metabolism, anti-inflammatory effects and immune regulation. It exists as D3, synthesized in the skin from 7-dehydrocholesterol via UVB, and as D2, found in some fungi. Both are hydroxylated in the liver to 25(OH)D and in the kidneys to the active 1.25(OH)_2_D [7,8]. Vitamin D receptors and enzymes are present in immune cells, supporting barrier and antimicrobial functions and possibly reducing allergy risk [8,9]. Variants in CYP2R1, GC, and VDR influence vitamin D levels and immune responses [10,11,12,13,14]. VDR is present in over 37 tissues and regulates 3000+ genes [15]. Environmental and genetic factors, which account for 23–80% of circulating 25(OH)D variation, shape vitamin D status and the response to deficiency [10,13,15,16,17].

Since the early 20th century, vitamin D deficiency has increased. Deficiency is classified as being below 20 ng/mL, with 20–30 ng/mL considered insufficient [11,18]. It is common in the US and Europe, varying by region, age, and ethnicity, with children and older adults often having lower levels [18]. A Turkish study found about one-third had severe deficiency (<10 ng/mL), nearly half had insufficiency (10–24 ng/mL), a smaller portion were normal (24–80 ng/mL), and 1–2% had high levels (>80 ng/mL) [19].

Vitamin A is vital for vision, cell development, and immunity. Its active form, retinoic acid, modulates and supports immune functions, promotes tolerance, activates immune cells, and maintains immune balance by increasing TGF-β. Deficiency impairs immunity and raises infection risk, especially in developing countries, affecting about a third of children and 250 million globally at risk. In Western countries like the UK, many young adults also have low vitamin A intake [20,21,22,23].

Vitamin A mainly exists as retinyl esters (RET) in animal foods and as provitamin A (proVA) carotenoids in plant foods. Most dietary vitamin A is retinyl palmitate, with β-carotene being the main proVA source. In developed countries, about 65% of vitamin A comes from preformed vitamin A, and 35% from proVA carotenoids (β-carotene 86%, α-carotene 10%, β-cryptoxanthin 4%) [20]. Both are converted into active metabolites like retinol, retinal, and retinoic acid. The bioavailability and conversion efficiency vary among individuals, largely due to genetic differences, especially in genes like Beta-Carotene Monooxygenase 1 (BCMO1) [20,21,22,24,25,26].

Vitamin D’s anti-inflammatory effects depend on calcitriol binding to the vitamin D receptor (VDR). The VDR–calcitriol complex then binds to RXR, activated by retinoic acid (RA), a vitamin A metabolite. This RXR-RA/VDR-D heterodimer regulates gene expression, including genes involved in inflammation and autoimmunity, by suppressing pro-inflammatory factors like NF-κB and reducing inflammatory mediators [27,28]. This mechanism enables vitamin D to modulate immune responses, suppress allergies, and decrease allergy mediators [3].

In allergic disease development, disruption of immune tolerance and chronic inflammation are key drivers, influenced by the environment, nutrition, and genetics [3,8,20]. Vitamins D and A help suppress inflammation by maintaining barriers and regulating immune cells [3,8,21]. Deficiencies are linked to allergies such as atopy, rhinitis, and dermatitis [3,8], and gene polymorphisms (CYP2R1, VDR, GC, BCMO1) affect vitamin metabolism and immune responses [8,20]. Studying vitamin A and D deficiencies aids understanding of the genetic factors in allergy risk [3,8,20,21].

This study aims to explore the relationships among serum vitamin A and 25(OH)D This study explores the relationships between serum vitamin A and 25(OH)D levels, selected genetic variants (*CYP2R1*, *GC*, *VDR*, and *BCMO1*), and allergy-related phenotypes. It examines whether genetic variation is associated with differences in vitamin status and allergy-related phenotypes. Given the retrospective design and limited sample size, the findings should be considered preliminary and hypothesis-generating, providing a basis for further investigation in larger prospective cohorts.

## 2. Materials and Methods

This retrospective study was conducted in accordance with the principles of the Declaration of Helsinki and was approved by the Scientific Research Ethics Committee of Istanbul Nisantasi University (Date: 3 November 2025, Approval No: 2025-08-03).

Participants were identified through a retrospective data analysis of individuals who had undergone genetic testing within the Epigenetic Coaching system and for whom serum vitamin A and vitamin D measurements were available. Participant criteria included individuals who had not taken any vitamin supplements (specifically vitamins A and D) in the previous three months and whose serum 25(OH)D concentrations were measured between November and February. Genetic data related to vitamin A and vitamin D metabolism were analyzed, and a total of 80 individuals were included in the study, comprising two groups of 40 participants each: one group with allergy-related phenotypes (eczema, *n* = 25, 62.5%; allergic rhinitis, *n* = 15, 37.5%) and one group without such symptoms. We classified the group with allergy-related phenotypes as the patient group, and individuals without allergy-related phenotypes as the control group. The patient (allergy-related phenotypes) group consisted of individuals exhibiting specific clinical symptoms of allergic rhinitis and/or eczema. Individuals without specific clinical symptoms of allergic rhinitis or eczema were classified as the control group.

In this study, serum vitamin A and 25(OH)D concentrations were evaluated separately in both groups and compared between patients and controls. Vitamin D status was defined according to serum 25-hydroxyvitamin D [25(OH)D] concentrations, with levels < 20 ng/mL classified as vitamin D deficiency and levels between 20 and 30 ng/mL classified as vitamin D insufficiency, in line with established clinical guidelines and previous epidemiological studies [11,18]. Serum vitamin A (retinol) levels < 1.2 µmol/L (approximately 343.7 µg/L) were considered low [29]. These cut-off values were used to categorize participants and to evaluate the association between vitamin status, allergic-like symptoms, and relevant genetic variants.

In addition, genetic polymorphisms related to the metabolism of vitamins A and D were examined, the distribution of these variants between the two groups was assessed, and potential correlations between the genetic polymorphisms and serum vitamin A and 25(OH)D concentrations were investigated.

### 2.1. Inclusion and Exclusion Criteria

The inclusion criteria for this study were defined as follows: having previously undergone genetic testing within the Epigenetic Coaching system for polymorphisms in the *CYP2R1*, *GC*, *VDR*, and *BCMO1* genes related to vitamin A and vitamin D metabolism; availability of serum vitamin A and 25(OH)D concentrations; and documented assessment of allergy-related phenotypes (present/absent). Exclusion criteria were as follows: incomplete data on vitamin A or 25(OH)D concentrations or genetic test results; lack of information regarding allergy-related phenotypes status; the presence of autoimmune diseases, chronic infectious diseases, chronic inflammatory disorders, chronic systemic diseases (including diabetes mellitus and cardiovascular diseases), known genetic disorders, or any other condition that could potentially influence vitamin A or vitamin D metabolism.

### 2.2. Genetic Analysis

Genomic DNA was isolated from buccal swab samples using the phenol–chloroform extraction method. The concentration and purity of the isolated DNA were assessed using a Nanodrop™ 1000 spectrophotometer (Thermo Scientific, Wilmington, DE, USA), and samples were normalized to a final concentration of 50 ng/μL.

Genotyping was performed using a custom-designed Infinium HTS iSelect microarray (Illumina, San Diego, CA, USA) following the manufacturer’s standard instructions. Briefly, 200 ng of genomic DNA was processed using the Infinium HD Super Assay Kit (Illumina). The procedure involved non-specific whole-genome amplification, followed by enzymatic fragmentation, precipitation, and resuspension. The fragmented DNA was hybridized onto the custom BeadChips at 48 °C for 16–24 h in an Illumina Hybridization Oven. Post-hybridization, unhybridized DNA was removed using Infinium Wash Buffers, and the chips underwent single-base extension and multi-layer staining using the Infinium HD Secondary Staining Kit (Illumina) to incorporate fluorescent labels. The processed BeadChips were imaged using the Illumina iScan system. SNV allele calls were generated using Illumina GenomeStudio software (version 2.0.5). All custom-designed markers were pre-validated for reproducibility and compared against reference materials. Samples with a call rate of <99% were excluded to ensure high-density data integrity. Sex-linked markers were verified for consistency with the reported gender of each individual [30]. The polymorphisms investigated in the vitamin D pathway included VDR rs1544410 (BsmI), rs2228570 (FokI), rs731236 (TaqI), and rs7975232 (ApaI); CYP2R1 rs1562902 and rs2060793; and GC rs2282679 and rs7041. For the vitamin A pathway, the investigated BCMO1 polymorphisms were rs11645428, rs12934922, rs6420424, rs6564851, and rs7501331. These polymorphisms were subsequently evaluated in relation to vitamin deficiency status and allergy-related phenotypes.

### 2.3. Statistical Analysis

Statistical analysis of the study data was performed using R software (version 4.5.1; R Foundation for Statistical Computing, Vienna Austria). The distribution characteristics of continuous variables were evaluated using visual (histogram and Q-Q plots) and analytical methods (Shapiro–Wilk normality test). For continuous variables that did not meet the normal distribution assumption, the non-parametric Mann–Whitney U test was used to compare them between two groups.

Serum vitamin A and 25(OH)D concentrations were converted into binary categorical variables deficiency present/absent according to clinical threshold values. Therefore, logistic regression analysis was used to evaluate genetic and demographic factors that may be associated with vitamin deficiency. Logistic regression analysis was preferred because the dependent variable has a binary structure and it allows for the control of potential confounding variables such as age, gender, and group within the same model.

Additive genetic modeling was applied to each variant to assess the association between genetic polymorphisms and vitamin deficiency. Analysis results were reported using odds ratios (ORs), 95% confidence intervals (CIs), and *p*-values.

The Chi-square test (Pearson χ^2^ test) was used to compare categorical variables between groups. This test evaluated whether the distributions of genotypes and alleles differed between groups.

Hardy–Weinberg equilibrium (HWE) was assessed for each genetic variant in the control group using an exact HWE test. A *p*-value > 0.05 was considered to indicate no statistically significant deviation from HWE.

In addition, the Apriori algorithm was applied in association rule mining to identify multiple genetic variant associations that may affect vitamin A and D deficiencies. In this analysis, genetic polymorphisms were recategorized based on the presence of risk alleles, and analyses were conducted separately for vitamin A and vitamin D deficiencies to identify which variant combinations are associated with deficiency states. Association rules were evaluated and reported based on support, confidence, and lift measures.

## 3. Results

In all statistical analyses, two-sided *p*-values were calculated, and *p* < 0.05 was considered statistically significant.

The study population comprised 80 participants, divided into the Allergy-related phenotypes group (*n* = 40) and the Control group (*n* = 40). The mean age of the Allergy-related phenotypes group was 33.58 ± 16.31 years, whereas the mean age of the Control group was 45.00 ± 14.44 years. When examined by age categories, participants in the Allergy-related phenotypes group were more evenly distributed across the 0–20, 21–40, and 41–60 age ranges, with no individuals older than 60 years. In contrast, in the Control group, the proportion of participants aged 41–60 years was markedly higher (62.5%), and 10.0% were aged 61 years and older.

The study population comprised 80 participants, divided into the group with allergy-related phenotypes (*n* = 40) and the control group (*n* = 40). The mean age of participants with allergy-related phenotypes was 33.58 ± 16.31 years, whereas the mean age of the control group was 45.00 ± 14.44 years. When examined by age categories, participants with allergy-related phenotypes were more evenly distributed across the 0–20, 21–40, and 41–60 year age groups, with no participants older than 60 years. In contrast, 62.5% of the control group were aged 41–60 years, while 10.0% were aged 61 years or older. Given this between-group age imbalance, age and sex were included as covariates in the subsequent multivariable analyses.

Gender distribution was similar across the two groups, with female participants comprising the majority. In the allergy-related phenotypes group, 70.0% of participants were female and 30.0% were male, while in the Control group, 77.5% were female and 22.5% were male. Although women predominated in both groups, the proportions were balanced and did not differ notably.

Overall, the demographic data indicate that the allergy-related phenotypes group consisted of a younger population, whereas the Control group was composed primarily of middle-aged and older individuals. Gender distribution was comparable across the two groups, with women constituting the majority in each. Detailed demographic characteristics are presented in Table 1.

### 3.1. Hardy–Weinberg Equilibrium Analysis

All investigated genetic variants were in Hardy–Weinberg equilibrium in the control group (all *p* > 0.05), with HWE *p*-values ranging from 0.152 to 1.000 (Table 2).

### 3.2. Comparison of Vitamin Levels Between Allergic Rhinitis and Eczema Subgroups

A subgroup analysis was performed by separating patients with allergic rhinitis and eczema. Serum 25(OH)D concentrations were 32.64 ± 13.57 ng/mL in patients with allergic rhinitis and 37.27 ± 16.22 ng/mL in patients with eczema, with no statistically significant difference between the groups (*p* = 0.428). Likewise, serum vitamin A levels did not differ significantly, with mean values of 464.90 ± 161.87 µg/L in the allergic rhinitis group and 383.97 ± 123.68 µg/L in the eczema group (*p* = 0.117) (Table 3).

### 3.3. Logistic Regression Analysis

In the multivariable logistic regression analyses, age, gender, and group were included as covariates together with the respective genetic variants. None of these covariates showed a statistically significant association with the categorical vitamin D or vitamin A deficiency outcomes in the logistic regression models (*p* > 0.05; Table 4). These findings are specific to the categorical deficiency outcomes evaluated in the logistic regression models and should not be interpreted as indicating an absence of associations with continuous serum vitamin concentrations.

### 3.4. Comparison of Serum Vitamin A and 25(OH)D Concentrations Between Groups

**Figure 1:**
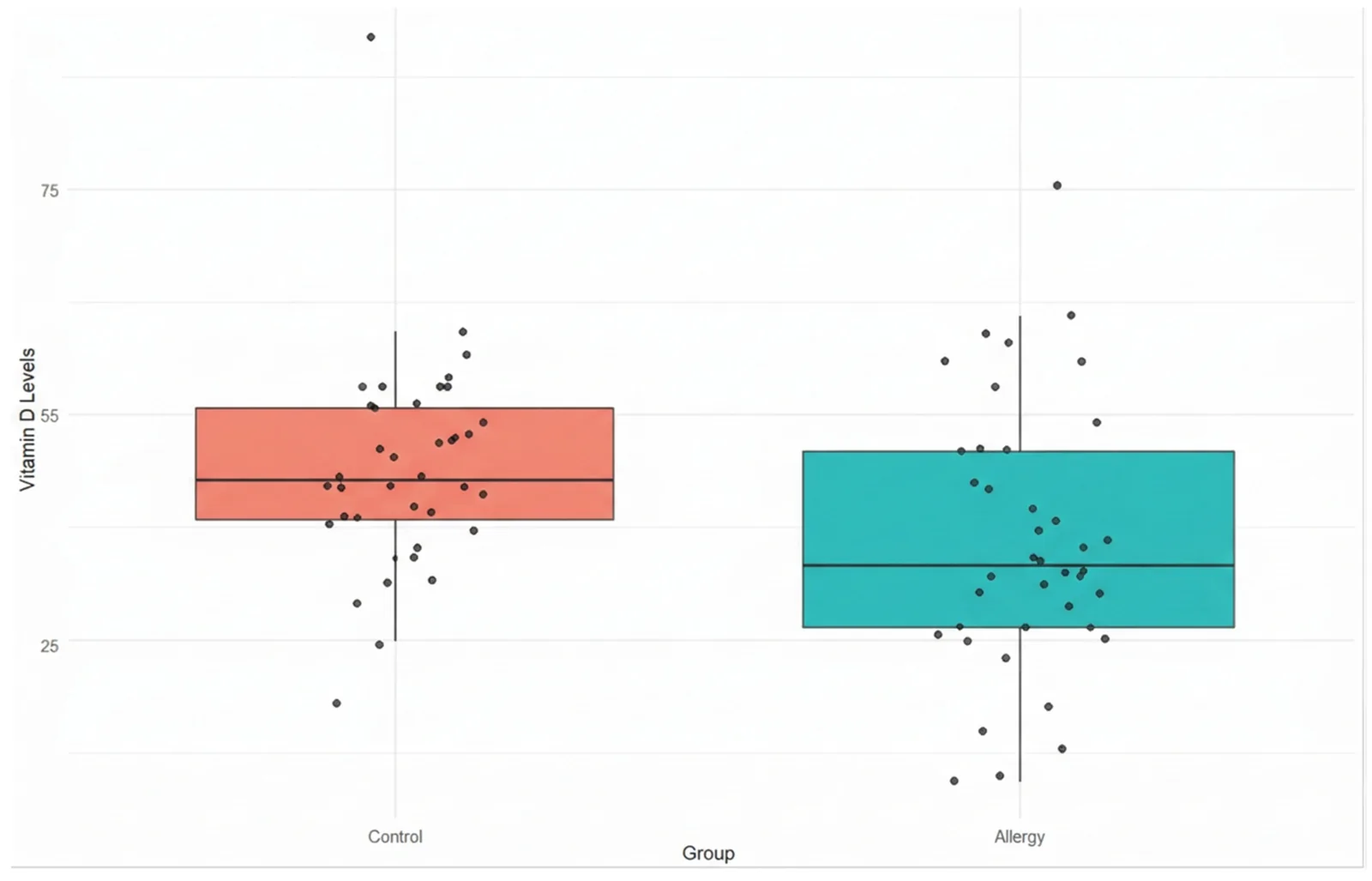
Boxplot Comparison of Serum Vitamin D Levels Between Allergy-related phenotypes and Control Groups.

Serum vitamin A and 25(OH)D concentrations were compared between the allergy-related phenotypes and control groups. For 25(OH)D concentrations, the assumption of normal distribution was not met, so the Mann–Whitney U test was used to compare the groups. The analysis revealed a statistically significant difference in serum 25(OH)D concentrations between the allergy-related phenotypes and control groups (Table 5a and Figure 1).

**Figure 2:**
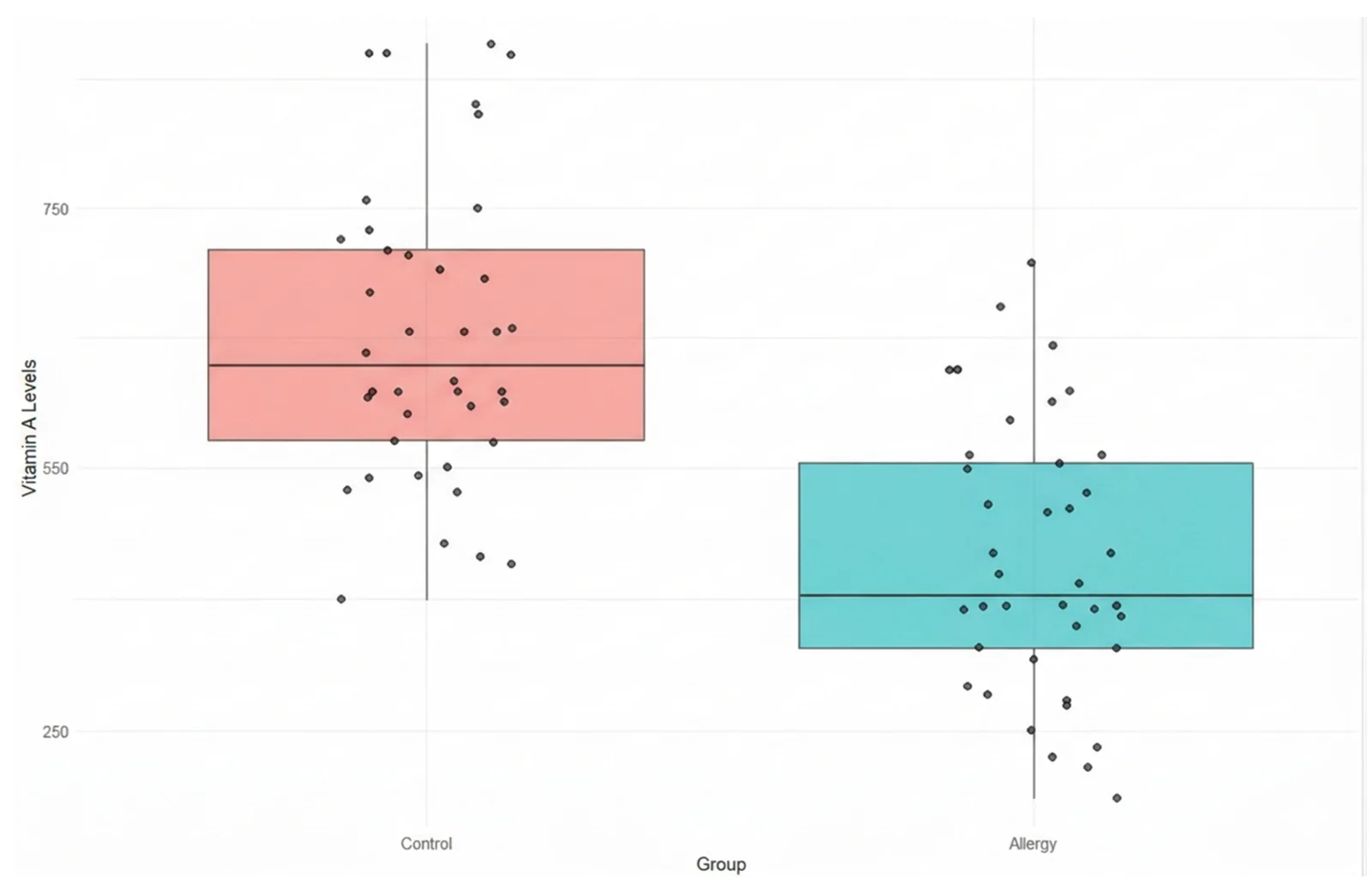
Boxplot Comparison of Serum Vitamin A Levels Between Allergy-related phenotypes and Control Groups.

The normality of vitamin A levels was assessed separately in the allergy-related phenotypes and control groups. Because the assumption of normality was met in both groups, an independent *t*-test was used to compare vitamin A levels between the groups. Accordingly, the mean vitamin A level in the control group was significantly higher than that in the allergy-related phenotypes group (Table 5b and Figure 2).

### 3.5. Multivariable Linear Regression Analysis of Factors Associated with Serum Vitamin D and Vitamin A Levels

To determine whether the observed differences in serum vitamin A and 25(OH)D concentrations were influenced by age, multivariable linear regression analyses were performed, adjusting for age and sex. As shown in (Table 6), the study group remained an independent predictor of both serum 25(OH)D (β = −7.48, 95% CI: −13.90 to −1.06, *p* = 0.023) and serum vitamin A concentrations (β = −204.53, 95% CI: −269.01 to −140.06, *p* < 0.001). In contrast, age was not independently associated with either serum 25(OH)D (*p* = 0.563) or vitamin A levels (*p* = 0.062). Female sex was independently associated with lower serum vitamin A levels (β = −80.86, 95% CI: −152.32 to −9.41, *p* = 0.027), whereas no significant association was observed with serum 25(OH)D concentrations (*p* = 0.986).

### 3.6. Genetic Comparisons in the Vitamin A and Vitamin D Panels

Analyses of the CYP2R1 rs2060793 polymorphism revealed a significant difference in genotype distributions between the allergy-related phenotypes and control groups (raw *p* = 0.005). The GG genotype was more frequent in the control group, whereas the AA genotype was more common in the allergy-related phenotypes group. After adjustment for multiple comparisons using the Benjamini–Hochberg false discovery rate (FDR) procedure, the association remained at the threshold of statistical significance (FDR-adjusted *p* = 0.048) (Table 7).

Similarly, the BCMO1 rs12934922 polymorphism showed a significant difference in genotype distribution between the allergy-related phenotypes and control groups before correction (raw *p* = 0.013). The AT genotype was more prevalent in the allergy-related phenotypes group, whereas the AA genotype was more frequent in the control group. However, after FDR correction, this association was no longer statistically significant (FDR-adjusted *p* = 0.081) (Table 7).

### 3.7. Association Rules

#### 3.7.1. Vitamin D Panel

In the exploratory association rule mining analysis, vitamin D deficiency was observed more frequently than expected for several genotype combinations within the study sample. Individuals carrying both *GC* rs2282679-AA and *VDR TAQ1* rs731236-TT genotypes had a 95.5% probability of vitamin D deficiency, and this combination was associated with an approximately 1.23-fold higher relative frequency of vitamin D deficiency in the sample (lift = 1.232). Similarly, individuals carrying both *VDR BSM1* rs1544410-GG and rs2282679-AA genotypes had a 94.7% probability of vitamin D deficiency, representing an approximately 1.22-fold higher relative frequency compared to the overall sample frequency (lift = 1.222) (Table 8).

Vitamin D deficiency was observed with a 92.9% probability among individuals with the *GC* rs2282679-AA genotype and *VDR APA1* rs7975232-CC, and this combination was associated with an approximately 1.20-fold higher relative frequency of vitamin D deficiency in the sample (lift = 1.198). The co-occurrence of *VDR* gene polymorphisms showed a similar trend. Vitamin D deficiency was detected with a 92.9% probability in individuals carrying both *VDR FOK1* rs2228570-AG and *VDR TAQ1* rs731236-TT genotypes, indicating an approximately 1.20-fold higher relative frequency of vitamin D deficiency in the sample (lift = 1.198) (Table 8).

Furthermore, vitamin D deficiency was observed with a 92–93% probability in individuals carrying combinations of *VDR FOK1* rs2228570-GG and *GC* rs7041-GG, as well as *VDR BSM1* rs1544410-GG and *VDR FOK1* rs2228570-AG, representing an approximately 1.19-fold higher relative frequency compared to the overall sample (lift = 1.191). Vitamin D deficiency was observed with a 91.7% probability in the combination of *GC* rs7041-GG and *VDR TAQ1* rs731236-TT, resulting in an approximately 1.18-fold higher relative frequency of vitamin D deficiency in the overall sample (lift = 1.183). Similarly, vitamin D deficiency was detected with a 91.7% probability in individuals with the *CYP2R1* rs1562902-CT genotype in combination with *GC* rs7041-TT, and this combination was associated with an approximately 1.18-fold higher relative frequency than expected in the sample (lift = 1.183) (Table 8).

#### 3.7.2. Vitamin A Panel

In the exploratory association rule mining analysis of pairwise BCMO1 genotype combinations, vitamin A deficiency was observed more often than expected for several genotype combinations in the study sample. Individuals carrying both *BCMO1* rs6420424-AA and *BCMO1* rs6564851-GG genotypes had a 50% probability of vitamin A deficiency, indicating that this association was approximately 2.2 times more frequent than expected in the sample (support = 0.050; lift = 2.222). Similarly, individuals carrying the *BCMO1* rs11645428-GG and *BCMO1* rs6564851-GG combination had a 60% probability of vitamin A deficiency, approximately 2.7 times the sample mean (support = 0.075; lift = 2.667) (Table 9).

Stronger associations were observed in some genotype combinations. Vitamin A deficiency was observed in all individuals carrying both *BCMO1* rs6564851-GG and *BCMO1* rs7501331-CC genotypes (confidence = 100%), indicating that this combination was approximately 4.4 times more frequent than the overall sample size for vitamin A deficiency (support = 0.075; lift = 4.444). Similarly, vitamin A deficiency was detected with a 66.7% probability in the combination of *BCMO1* rs6420424-AA and *BCMO1* rs7501331-CC, indicating that the deficiency was approximately 3 times more frequent in the sample (support = 0.050; lift = 2.963) (Table 9).

Furthermore, individuals carrying both *BCMO1* rs12934922-TT and *BCMO1* rs6420424-AG genotypes had a 66.7% probability of vitamin A deficiency, and this combination was associated with approximately 3-fold higher relative frequency of vitamin A deficiency in the sample (support = 0.050; lift = 2.963) (Table 9).

## 4. Discussion

Vitamin D is a critical immunoregulatory molecule that influences both innate and adaptive immune pathways, including epithelial barrier integrity, T-cell differentiation, and antimicrobial peptide synthesis [8,9]. Consistent with the extensive body of literature demonstrating an association between vitamin D insufficiency and increased allergy risk particularly in conditions such as atopy, asthma, and allergic rhinitis [3,5,6,8,9,11] our study likewise found that serum 25(OH)D concentrations were significantly lower in the allergic group compared with the control group (Table 5a).

The *CYP2R1* gene, a key enzyme in vitamin D metabolism, has been identified as an important genetic determinant of allergy susceptibility. Previous studies have shown that the rs2060793 variant, particularly the AA genotype, is associated with an increased risk of allergy in younger individuals and may create a genetic predisposition related to age [32]. Consistent with these findings, in our study, the genotype distribution of the *CYP2R1* rs2060793 polymorphism also differed significantly between the allergy-related phenotypes and control groups (*p* = 0.005), with the AA genotype more frequently observed in the allergy-related phenotypes group (Table 7).

Although the *CYP2R1* rs1562902 variant did not show a significant independent association in our study, earlier studies have suggested that variants in *CYP2R1* rs1562902 may contribute to interindividual differences in vitamin D status [10,15]. Consistent with this possibility, association rule mining analysis of our dataset showed that rs1562902, when evaluated together with the *GC* rs7041 variant, was associated with vitamin D deficiency with high confidence and higher-than-expected relative frequency. Specifically, vitamin D deficiency was detected with a 91.7% probability in individuals carrying the *CYP2R1* rs1562902-CT genotype together with *GC* rs7041-TT, and this combination was associated with an approximately 1.18-fold higher relative frequency than expected in the sample (Table 8).

The *GC* gene is another important genetic determinant influencing vitamin D bioavailability. In particular, the rs7041 polymorphism has been shown to modulate the relationship between serum vitamin D levels and allergy risk. This variant affects DBP (vitamin D–binding protein) concentrations, with individuals carrying the GT/TT genotypes exhibiting a tendency toward lower DBP levels. Reduced DBP levels can influence the circulating free fraction of 25(OH)D and thereby affect the regulation of immune responses. Moreover, studies reporting that maternal vitamin D supplementation reduces the risk of food allergy especially in infants with the GT/TT genotype further underscore the clinical relevance of this gene–environment interaction [33]. In another study, the rs2282679 genetic variant of the *GC* gene was shown to be associated with low circulating 25-hydroxyvitamin D levels [33,34].

In our study, association rule mining further supported the relevance of *GC* variants by showing that both *GC* rs2282679 and *GC* rs7041 appeared repeatedly in genotype combinations associated with vitamin D deficiency. Specifically, *GC* rs2282679 formed high-confidence combinations with *VDR TAQ1* (95.5%), *VDR BSM1* (94.7%), and *VDR APA1* (92.9%), while *GC* rs7041 was involved in combinations with *VDR FOK1* (92.3%), *VDR TAQ1* (91.7%), and *CYP2R1* rs1562902 (91.7%). Taken together, these findings suggest that *GC* variants may play a more meaningful role in vitamin D deficiency when evaluated in combination with polymorphisms involved in vitamin D receptor signaling and metabolic activation, rather than in isolation (Table 8).

Polymorphisms in the *VDR* are well established in the literature as important factors influencing both vitamin D metabolism and susceptibility to allergic diseases. Variants such as *FokI*, *BsmI*, *ApaI*, and *TaqI* have been shown to affect a range of immune processes, including serum 25(OH)D concentrations, response to supplementation, T-cell differentiation, and cytokine production; therefore, these variants have been associated with asthma, atopic dermatitis, and other atopic conditions [4,35,36,37,38]. Although meta-analyses do not consistently support an association for rs2228570 *FOK1,* they indicate that variants such as rs7975232 *APA1* (A), rs1544410 *BSM1* (G), and rs731236 *TAQ1* (T) may increase allergy risk in certain populations [4].

Consistent with this body of evidence, our findings suggest that *VDR* polymorphisms (*TAQ1*, *BSM1*, *FOK1*, and *APA1*), particularly when present in combination with *GC* gene variants or with one another, may contribute to susceptibility to vitamin D deficiency. The high probability and higher-than-expected relative frequency observed for these pairwise genotype combinations in the overall sample further support the relevance of *VDR* polymorphisms in vitamin D metabolism and signal transduction. In particular, the likelihood of vitamin D deficiency in individuals carrying the *VDR FOK1* rs2228570-AG and *VDR TAQ1* rs731236-TT genotype combination was found to be 92.9% in the sample. Similarly, the probability of vitamin D deficiency in individuals with both the *VDR FOK1* rs2228570-AG and *VDR BSM1* rs1544410-GG genotypes was also found to be 92.3% (Table 8).

Our findings indicate that a deficiency in vitamin D may be linked to concurrent variations in genes associated with vitamin D binding, transport, metabolism, and receptor-level signaling. Exploratory association rule mining revealed multi-variant co-occurrence patterns within the study sample; however, these results should not be construed as definitive evidence of gene–gene interactions or independent genetic effects. They are preliminary and hypothesis-generating, necessitating validation in larger prospective cohorts.

Genetic variants also play a critical role in determining the efficacy and safety of high-dose vitamin D supplementation. For example, individuals carrying *CYP24A1* mutations may have an increased risk of kidney stone formation when taking vitamin D [39]. Understanding these genetic influences may facilitate the development of more personalized and effective nutritional interventions.

Vitamin A plays a role at least as essential as that of vitamin D in mucosal immunity, epithelial differentiation, and the development of regulatory T cells [20,21]. Consistent with the literature reporting increased infectious and inflammatory risk in the context of vitamin A deficiency [21,22,23], our study found that serum vitamin A levels were significantly lower in the allergic group than in the control group (Table 5b). *BCMO1* is the key enzyme responsible for converting provitamin A carotenoids into retinoids and is expressed not only in intestinal enterocytes but also in many tissues with high vitamin A demand. Although up to 95% of β-carotene can theoretically be converted into retinoids, this efficiency varies substantially among individuals due to polymorphisms in the *BCMO1* gene [21,22,23].

Among the genetic variants involved in vitamin A metabolism, *BCMO1* rs6564851 is one of the best characterized. The presence of the G allele has been associated with higher β-carotene levels. This variant has been reported to reduce *BCMO1* activity by up to 90%, potentially leading to hypercarotenemia and hypovitaminosis A [40]. For the rs11645428 variant, conversion efficiency in GG homozygotes is 51% lower than in individuals carrying the AA genotype. Similarly, in the rs6420424 variant, AA homozygotes exhibit a 59% reduction in conversion efficiency relative to GG carriers [23].

Another study demonstrated that higher intake of preformed vitamin A reduces the risk of asthma onset and improves lung function. The relationship between vitamin A intake and pulmonary function is modified by *BCMO1* polymorphisms. Preformed vitamin A intake (rather than β-carotene) was associated with reduced risk of asthma onset, airway obstruction, and impaired pulmonary function in children. For the *BCMO1* rs7501331 variant, carriers of the T allele show lower β-carotene conversion efficiency compared with C allele carriers; for the rs12934922 variant, the A allele is associated with higher conversion rates. Notably, the TT genotype at rs12934922 has been associated with an increased risk of asthma among individuals with high β-carotene intake [41].

In our study, the *BCMO1* rs12934922 polymorphism exhibited a nominally significant difference in genotype distribution between the group with allergy-related phenotypes and the control group (*p* = 0.013). The AT genotype was found more frequently in the allergy-related phenotypes group, whereas the AA genotype was more prevalent in the control group (Table 7); nevertheless, this association did not retain statistical significance after correction for multiple testing using the False Discovery Rate (FDR) and should therefore be regarded as exploratory and hypothesis-generating rather than as evidence of an established or causal genetic effect.

Collectively, these findings imply that variability within the *BCMO1* pathway may be linked to vitamin A status; nonetheless, the current data do not substantiate an independent influence of any singular *BCMO1* variant on allergy-related phenotypes. Exploratory association rule mining revealed co-occurrence patterns involving multiple *BCMO1* variants that were correlated with vitamin A deficiency. Specifically, the combinations rs6420424–rs6564851 and rs11645428–rs6564851 were associated with approximately 2.2- and 2.7-fold higher incidences of vitamin A deficiency, respectively, whereas rs6564851–rs7501331 and rs6420424–rs7501331 demonstrated approximately 4.4- and 3-fold increased occurrences. Additionally, the combination rs12934922–rs6420424 was linked to roughly a 3-fold higher incidence of vitamin A deficiency (see Table 9). It is imperative to note that these findings reflect multi-variant co-occurrence patterns rather than the independent effects of individual single nucleotide polymorphisms (SNPs), and should be considered exploratory and hypothesis-generating.

Although vitamin A has been reported to exert protective effects against certain allergic conditions by suppressing inflammatory responses [3], the literature is not entirely consistent. Some studies have shown that predisposition to atopy may increase, particularly among young women, and that early-life supplementation (including vitamin A) may elevate asthma risk in infants and increase the likelihood of food allergy in formula-fed infants [42,43]. These conflicting results suggest that the factors determining whether vitamin A supplementation is beneficial or potentially harmful vary substantially with individual characteristics, including age, genetic background, timing of supplementation, mode of administration, and baseline nutritional status.

An increasing body of research demonstrates that vitamins A and D function in an interdependent manner to regulate immune responses. The *VDR*–RXR heterodimer, which is essential for vitamin D–mediated transcription, is activated by retinoic acid, the active metabolite of vitamin A. Therefore, a deficiency or genetic disruption in either pathway may adversely affect anti-inflammatory signaling, epithelial barrier repair, or regulatory T-cell differentiation. This mechanism indicates that the anti-inflammatory effects of vitamin D require the formation of an active heterodimer and thus depend on the presence of vitamin A. For this reason, co-supplementation of vitamins D and A has been recommended in practical applications [27].

Our study found lower serum vitamin A and 25(OH)D levels in those with allergy-related phenotypes, suggesting a possible link. Of the genetic variants examined, only *CYP2R1* rs2060793 remained significant after FDR correction; the result for *BCMO1* rs12934922 was exploratory. Additionally, association rule mining revealed co-occurring patterns involving *GC, VDR, CYP2R1,* and *BCMO1* variants associated with vitamin deficiencies. These patterns are preliminary and hypothesis-generating, not proof of genetic susceptibility or causality. Findings indicate that lower vitamin A and D levels in those with allergy-related phenotypes may relate to environmental factors and genetic variation in vitamin metabolism. Association rule mining revealed multi-variant patterns linked to deficiencies, but not as evidence of genetic burden or interactions. The coordinated action of vitamin A and D pathways via RXR/VDR heterodimerization offers a plausible mechanism for immune regulation, which needs further study, as it was not directly examined.

Although vitamin D deficiency has frequently been associated with allergic diseases, the relationship appears to be more complex than previously thought. Several studies have suggested a possible U-shaped association, with both low and high serum 25(OH)D concentrations being linked to an increased risk of allergic sensitization or atopy [44]. Therefore, maintaining optimal vitamin D status, rather than excessive supplementation, may be more appropriate. However, further well-designed prospective studies are needed to clarify this relationship.

This study presents an exploratory evaluation of the associations among genetic variants implicated in vitamin A and vitamin D metabolism, vitamin status, and allergy-related phenotypes. To the best of our knowledge, few investigations have concurrently examined *BCMO1* variants associated with vitamin A metabolism alongside *CYP2R1*, *VDR*, and *GC* variants involved in vitamin D metabolism within this context. The research compares serum levels of vitamin A and 25(OH)D between individuals with allergy-related phenotypes and control subjects, and analyzes genotype distributions as well as exploratory co-occurrence patterns of multiple variants. By integrating biochemical and genetic data, this methodology offers a comprehensive descriptive framework for examining potential genotype–phenotype correlations. Nonetheless, considering the retrospective nature of the study and the limited sample size, these results should be regarded as preliminary and hypothesis-generating, and not as evidence of definitive genetic susceptibility, causal relationships, or direct implications for personalized treatment approaches.

## 5. Limitations

Several limitations warrant acknowledgment. Firstly, the relatively modest sample size and the single-center, retrospective design may constrain the generalizability of the results. Additionally, the allergy-related phenotype group was younger than the control group (33.58 ± 16.31 vs. 45.00 ± 14.44 years). Although age and sex were incorporated as covariates in the multivariable analyses, the limited sample size does not entirely eliminate the possibility of residual confounding. Consequently, the differences observed between groups should be interpreted with caution. Moreover, environmental, lifestyle, dietary, and other potentially pertinent confounding factors were not systematically controlled, which is particularly significant in nutrigenetic research. Therefore, these findings necessitate validation in larger, prospective cohorts matched for age and sex.

Another significant limitation pertains to the retrospective classification of allergy-related phenotypes. Participants were categorized based on previously documented clinical diagnoses and allergy-related symptoms; however, objective confirmation of allergic sensitization—including skin-prick testing and/or serum-specific IgE measurements—was not consistently available. As a result, it was not feasible to reliably distinguish IgE-mediated sensitization from non-IgE-mediated conditions. Furthermore, the retrospective study design precludes causal inference. Additionally, considering the limited sample size and multiple genetic comparisons, the genetic findings should be regarded as preliminary and exploratory. Only CYP2R1 rs2060793 remained statistically significant after FDR correction, whereas the association for BCMO1 rs12934922 did not retain significance. Therefore, the present results should be viewed as hypothesis-generating and should not be interpreted as evidence of established genetic susceptibility, causal effects, individualized risk prediction, or direct clinical applicability.

## 6. Conclusions

Our study identified lower serum levels of vitamin A and 25(OH)D in individuals exhibiting allergy-related phenotypes, indicating a potential correlation between diminished vitamin statuses and these phenotypes. Additionally, the research uncovered preliminary associations between certain genetic variants implicated in vitamin A and vitamin D metabolism and allergy-related phenotypes. Among the variants examined, only *CYP2R1* rs2060793 maintained statistical significance following FDR correction; in contrast, the association with *BCMO1* rs12934922 did not persist and should thus be regarded as exploratory. Moreover, exploratory association rule mining revealed multi-variant co-occurrence patterns involving *GC*, *VDR*, *CYP2R1*, and *BCMO1* concerning vitamin A and D deficiencies. These findings may reflect multivariate co-occurrence patterns, but they do not constitute formal evidence of gene–gene interactions. Overall, the present findings are preliminary and hypothesis-generating and require validation in larger, prospectively designed, age- and sex-matched cohorts that incorporate objective assessments of allergic sensitization, comprehensive nutritional evaluation, appropriate correction for multiple testing, and functional analyses of relevant genetic variants before any clinical application, individualized risk assessment, preventive strategy, or personalized intervention can be considered.

## Figures and Tables

**Table 1:** Demographic Characteristics of the Study Population.

Variable	Allergy-Related Phenotypes (*n* = 40)	Control Group (*n* = 40)
Age (Mean ± SD)	33.58 ± 16.31	45.00 ± 14.44
Sex, *n* (%)		
Female	28 (70.0%)	31 (77.5%)
Male	12 (30.0%)	9 (22.5%)
Age groups, *n* (%)		
0–20	10 (25.0%)	4 (10.0%)
21–40	14 (35.0%)	7 (17.5%)
41–60	16 (40.0%)	25 (62.5%)
61+	0 (0.0%)	4 (10.0%)

SD, Standard Deviation; *n*, number of participants; %, percentage.

**Table 2:** Hardy–Weinberg Equilibrium Analysis of Genetic Variants in the Control Group.

Gene/SNP	Genotype Counts in the Control Group (*n*)	HWE *p*
VDR rs1544410	AA = 6, AG = 19, GG = 15	1.000
CYP2R1 rs1562902	CC = 5, CT = 17, TT = 18	0.732
CYP2R1 rs2060793	AA = 1, AG = 15, GG = 24	0.659
VDR rs2228570	AA = 4, AG = 18, GG = 18	1.000
GC rs2282679	AA = 24, AC = 13, CC = 3	0.659
GC rs7041	GG = 11, GT = 20, TT = 9	1.000
VDR rs731236	CC = 4, CT = 20, TT = 16	0.731
VDR rs7975232	AA = 10, AC = 19, CC = 11	0.758
BCMO1 rs11645428	AA = 2, AG = 23, GG = 15	0.152
BCMO1 rs12934922	AA = 18, AT = 18, TT = 4	1.000
BCMO1 rs6420424	AA = 10, AG = 19, GG = 11	0.758
BCMO1 rs6564851	GG = 9, GT = 21, TT = 10	1.000
BCMO1 rs7501331	CC = 21, CT = 14, TT = 5	**0.279**

**Table 3:** Independent Samples *t*-test Analysis.

Variable	Allergic Rhinitis (*n* = 15) Mean ± SD	Eczema (*n* = 25) Mean ± SD	*p*-Value
Vitamin D	33.12 ± 11.56	37.43 ± 16.49	0.381
Vitamin A	427.35 ± 150.88	390.48 ± 122.21	0.403

**Table 4:** Logistic Regression Analysis.

Gene	Risk Allele Frequency (RAF)	Dependent Variable	Independent Variables	OR	95% CI	*p*
*VDR (BSM1)*-rs1544410 G (risk allele)	0.62	Vitamin D	*VDR (BSM1)*-rs1544410	1.32	**0.61–2.85**	0.48
*CYP2R1*-rs1562902 T (risk allele)	0.56	Vitamin D	*CYP2R1*-rs1562902	0.81	**0.37–1.78**	0.60
			Age	0.99	**0.96–1.02**	0.53
			Gender	1.79	**0.43–7.37**	0.42
			Group	0.71	**0.22–2.25**	0.56
*CYP2R1*-rs2060793 G (risk allele)	0.62	Vitamin D	*CYP2R1*-rs2060793	0.97	**0.37–2.51**	0.95
			Age	0.98	**0.93–1.04**	0.47
			Gender	1.74	**0.43–7.10**	0.44
			Group	0.69	**0.21–2.26**	0.54
*VDR (FOK1)*-rs2228570 G (risk allele)	0.63	Vitamin D	*VDR (FOK1)*-rs2228570	1.96	**0.88–4.37**	0.10
			Age	0.98	**0.93–1.03**	0.46
			Gender	1.86	**0.44–7.89**	0.40
			Group	0.76	**0.22–2.58**	0.66
*GC*-rs2282679 C (risk allele)	0.26	Vitamin D	*GC*-rs2282679	0.53	**0.23–1.21**	0.13
			Age	0.99	**0.96–1.02**	0.52
			Gender	2.00	**0.45–8.82**	0.36
			Group	0.59	**0.18–1.96**	0.39
*GC*-rs7041 T (risk allele)	0.48	Vitamin D	*GC*-rs7041	1.01	**0.60–1.70**	0.97
			Age	0.98	**0.93–1.03**	0.46
			Gender	1.73	**0.42–7.17**	0.45
			Group	0.68	**0.21–2.20**	0.52
*VDR (TAQ1)*-rs731236 C (risk allele)	0.37	Vitamin D	*VDR (TAQ1)*-rs731236	0.56	**0.24–1.31**	0.18
			Age	0.98	**0.93–1.03**	0.46
			Gender	1.55	**0.37–6.52**	0.55
			Group	0.74	**0.23–2.43**	0.62
*VDR (APA1)*-rs7975232 C (risk allele)	0.46	Vitamin D	*VDR (APA1)*-rs7975232	0.98	**0.44–2.16**	0.96
			Age	0.98	**0.93–1.03**	0.46
			Gender	1.76	**0.41–7.63**	0.45
			Group	0.68	**0.22–2.14**	0.51
*BCMO1*-rs11645428 G (risk allele)	0.68	Vitamin A	*BCMO1*-rs11645428	2.50	**0.65–9.54**	0.18
			Age	1.01	**0.96–1.06**	0.70
			Gender	2.26	**0.45–11.27**	0.32
			Group	-	**-**	0.99
*BCMO1*-rs12934922 T (risk allele)	0.59	Vitamin A	*BCMO1*-rs12934922	0.91	**0.24–3.46**	0.89
			Age	1.01	**0.98–1.05**	0.57
			Gender	2.41	**0.50–11.50**	0.27
			Group	-	**-**	0.99
*BCMO1*-rs6420424 A (risk allele)	0.48	Vitamin A	*BCMO1*-rs6420424	2.16	**0.63–7.39**	0.22
			Age	1.02	**0.95–1.09**	0.57
			Gender	1.83	**0.36–9.43**	0.47
			Group	-	**-**	0.99
*BCMO1*-rs6564851 G (risk allele)	0.50	Vitamin A	*BCMO1*-rs6564851	3.19	**0.84–12.09**	0.088
			Age	1.02	**0.97–1.07**	0.41
			Gender	2.11	**0.43–10.44**	0.36
			Group	-	**-**	0.99
*BCMO1*-rs7501331 T (risk allele)	0.20	Vitamin A	*BCMO1*-rs7501331	0.58	**0.11–2.93**	0.51
			Age	1.02	**0.96–1.08**	0.51
			Gender	2.73	**0.53–14.07**	0.23
			Group	-	**-**	0.99

Abbreviations: OR, odds ratio; RAF, risk allele frequency. RAF values represent European population allele frequencies obtained from the NCBI Allele Frequency Aggregator (ALFA) database [31].

**Table 5:** (**a**). Comparison of Serum Vitamin D Levels Between Allergy-related phenotypes and Control Groups (Mann–Whitney U Test). (**b**). Comparison of Serum Vitamin A Levels Between allergy-related phenotypes and Control Groups (Independent *t*-test).

(**a**)					
**Variable**	**Group**	**W**		***p***	
25(OH)D	Control	1099.5		0.004	
	Allergy-related phenotypes				
(**b**)					
**Variable**	**Group**	**Mean**	**T**		***p***
Vitamin A	Control	624.24	7.00		<0.001
	Allergy-related phenotypes	404.31			

**Table 6:** Multivariable Linear Regression Analysis.

Outcome	Variable	β (95% CI)	*p*-Value
25(OH)D	Group (Allergy-related phenotypes vs. Control)	−7.48 (−13.90 to −1.06)	0.023
	Age (years)	0.06 (−0.15 to 0.28)	0.563
	Female sex	0.06 (−7.05 to 7.18)	0.986
Vitamin A	Group (Allergy-related phenotypes vs. Control)	−204.53 (−269.01 to −140.06)	<0.001
	Age (years)	2.07 (−0.10 to 4.25)	0.062
	Female sex	−80.86 (−152.32 to −9.41)	0.027

**Table 7:** Genetic Comparisons in the Vitamin A and Vitamin D Panel.

Gene	Risk Allele	Allergy-Related Phenotypes (*n*)	Control Group (*n*)	*p*	FDR-Adjusted *p*-Value
*CYP2R1*-rs2060793 G (Risk Allele)	AA	10	1	0.005	0.048
	AG	17	15		
	GG	13	24		
*BCMO1*-rs12934922 T (Risk Allele)	AA	6	18	0.013	0.081
	AT	27	18		
	TT	7	4		

**Table 8:** Vitamin D Panel–Binary LHS (Gene Association).

LHS (Gene Association)	RHS	Support	Confidence	Lift
*GC* rs2282679 (G)= AA *VDR TAQ1* (rs731236) (C) = TT	Vitamin D deficiency	0.263	0.955	1.232
*VDR BSM1* (rs1544410) (G) = GG *GC* rs2282679 (C) = AA	Vitamin D deficiency	0.225	0.947	1.222
*GC* rs2282679 (C) = AA *VDR APA1* (rs7975232) (C) = CC	Vitamin D deficiency	0.163	0.929	1.198
*VDR FOK1* (rs2228570) (G) = AG *VDR TAQ1* (rs731236) (C) = TT	Vitamin D deficiency	0.163	0.929	1.198
*VDR FOK1* (rs2228570) (G) = GG *GC* rs7041 (T) = GG	Vitamin D deficiency	0.150	0.923	1.191
*VDR BSM1* (rs1544410) (G) = GG *VDR FOK1* (rs2228570) (G) = AG	Vitamin D deficiency	0.150	0.923	1.191
*GC* rs7041 (T) = GG *VDR TAQ1* (rs731236) (C) = TT	Vitamin D deficiency	0.138	0.917	1.183
*CYP2R1* rs1562902 (T) = CT *GC* rs7041 (T) = TT	Vitamin D deficiency	0.138	0.917	1.183

Abbreviations: Support, proportion of observations containing the rule; Confidence, conditional probability of the consequent given the antecedent; Lift, measure of association strength between antecedent and consequent, where values >1 indicate a positive association.

**Table 9:** Vitamin A Panel–Binary LHS (Gene Association).

LHS (Gene Association)	RHS	Support	Confidence	Lift
*BCMO1* rs6420424 (A) = AA *BCMO1* rs6564851 (G)= GG	Vitamin A deficiency	0.050	0.500	2.222
*BCMO1* rs11645428 (G) = GG *BCMO1* rs6564851 (G) =GG	Vitamin A deficiency	0.075	0.600	2.667
*BCMO1* rs6564851 (G) = GG *BCMO1* rs7501331 (T) = CC	Vitamin A deficiency	0.075	1.000	4.444
*BCMO1* rs6420424 (A) = AA *BCMO1* rs7501331 (T) = CC	Vitamin A deficiency	0.050	0.667	2.963
*BCMO1* rs12934922 (T) = TT *BCMO1* rs6420424 (A) = AG	Vitamin A deficiency	0.050	0.667	2.963

Abbreviations: Support, proportion of observations containing the rule; Confidence, conditional probability of the consequent given the antecedent; Lift, measure of association strength between antecedent and consequent, where values >1 indicate a positive association.

## Data Availability

The data presented in this study are available on request from the corresponding author due to privacy and ethical restrictions.

## References

[1] Sánchez-Borges M., Martin B.L., Muraro A.M., Wood R.A., Agache I.O., Ansotegui I.J., Casale T.B., Fleisher T.A., Hellings P.W., Papadopoulos N.G. (2018). The importance of allergic disease in public health: An iCAALL statement. World Allergy Organ. J..

[2] Catak J., Demirci A., Yaman M. (2021). Food Allergies and Microbiota. Eur. J. Sci. Technol..

[3] Hufnagl K., Jensen-Jarolim E. (2018). Vitamin A and D in allergy: From experimental animal models and cellular studies to human disease. Allergo J. Int..

[4] Zhang L., Zhang S., He C., Wang X. (2020). VDR gene polymorphisms and allergic diseases: Evidence from a meta-analysis. Immunol. Investig..

[5] Ashok N., Kirubakaran R., Saraswathy R. (2020). Association of vitamin D gene polymorphisms in children with asthma: A systematic review. Heliyon.

[6] Ju F., Zhu R. (2023). Association of vitamin D levels and VDR variant (rs2228570) with allergic rhinitis: A meta-analysis and trial sequential analysis. Heliyon.

[7] Niforou A., Konstantinidou V., Naska A. (2020). Genetic variants shaping inter-individual differences in response to dietary intakes: A narrative review of the case of vitamins. Front. Nutr..

[8] Suaini N.H., Zhang Y., Vuillermin P.J., Allen K.J., Harrison L.C. (2015). Immune modulation by vitamin D and its relevance to food allergy. Nutrients.

[9] Szydziak J.M., Hrapkowicz A., Janowska K., Dąbkowska D., Szeidl O., Rehan D., Wołoszczak J., Mioskowska A. (2024). The role of vitamin D in allergic diseases. Qual. Sport.

[10] Bösch E.S., Spörri J., Scherr J. (2025). Vitamin metabolism and its dependency on genetic variations among healthy adults: A systematic review for precision nutrition strategies. Nutrients.

[11] Briceno Noriega D., Savelkoul H.F.J. (2021). Vitamin D and allergy susceptibility during gestation and early life. Nutrients.

[12] Uitterlinden A.G., Fang Y., Van Meurs J.B., Pols H.A., Van Leeuwen J.P. (2004). Genetics and biology of vitamin D receptor polymorphisms. Gene.

[13] Jolliffe D.A., Walton R.T., Griffiths C.J., Martineau A.R. (2016). Single nucleotide polymorphisms in the vitamin D pathway associating with circulating concentrations of vitamin D metabolites and non-skeletal health outcomes: Review of genetic association studies. J. Steroid Biochem. Mol. Biol..

[14] Ruiz-Ballesteros A.I., Meza-Meza M.R., Vizmanos-Lamotte B., Parra-Rojas I., de la Cruz-Mosso U. (2020). Association of vitamin D metabolism gene polymorphisms with autoimmunity: Evidence in population genetic studies. Int. J. Mol. Sci..

[15] Poon A.H., Gong L., Brasch-Andersen C., Litonjua A.A., Raby B.A., Hamid Q., Laprise C., Weiss S.T., Altman R.B., Klein T.E. (2012). Very important pharmacogene summary for VDR. Pharmacogenet. Genom..

[16] Żmijewski M.A. (2022). Nongenomic activities of vitamin D. Nutrients.

[17] Bahrami A., Sadeghnia H.R., Tabatabaeizadeh S.A., Bahrami-Taghanaki H., Behboodi N., Esmaeili H., Ferns G.A., Mobarhan M.G., Avan A. (2018). Genetic and epigenetic factors influencing vitamin D status. J. Cell. Physiol..

[18] Amrein K., Scherkl M., Hoffmann M., Neuwersch-Sommeregger S., Köstenberger M., Tmava Berisha A., Martucci G., Pilz S., Malle O. (2020). Vitamin D deficiency 2.0: An update on the current status worldwide. Eur. J. Clin. Nutr..

[19] Meral G., Guven A., Uslu A., Can G., Yozgatli A.U., Yaprak P., Akcay F. (2016). The prevalence of vitamin D deficiency in children, adolescents and adults in a sample of Turkish population. Stud. Ethno-Med..

[20] Borel P., Desmarchelier C. (2017). Genetic variations associated with vitamin A status and vitamin A bioavailability. Nutrients.

[21] Carazo A., Macáková K., Matoušová K., Krčmová L.K., Protti M., Mladěnka P. (2021). Vitamin A update: Forms, sources, kinetics, detection, function, deficiency, therapeutic use and toxicity. Nutrients.

[22] Graßmann S., Pivovarova-Ramich O., Henze A., Raila J., Ampem Amoako Y., King Nyamekye R., Bedu-Addo G., Mockenhaupt F.P., Schulze M.B., Danquah I. (2020). SNV rs6564851 in the BCO1 gene is associated with varying provitamin A plasma concentrations but not with retinol concentrations among adolescents from rural Ghana. Nutrients.

[23] Lietz G., Oxley A., Leung W., Hesketh J. (2012). Single nucleotide polymorphisms upstream from the β-carotene 15,15′-monoxygenase gene influence provitamin A conversion efficiency in female volunteers. J. Nutr..

[24] Zumaraga M.P.P., Arquiza J., Concepcion M.A., Perlas L., Alcudia-Catalma M.N., Rodriguez M. (2022). Genotype effects on β-carotene conversion to vitamin A: Implications on reducing vitamin A deficiency in the Philippines. Food Nutr. Bull..

[25] Lobo G.P., Amengual J., Baus D., Shivdasani R.A., Taylor D., von Lintig J. (2013). Genetics and diet regulate vitamin A production via the homeobox transcription factor ISX. J. Biol. Chem..

[26] Widjaja-Adhi M.A., Lobo G.P., Golczak M., von Lintig J. (2015). A genetic dissection of intestinal fat-soluble vitamin and carotenoid absorption. Hum. Mol. Genet..

[27] Riccio P., Rossano R. (2018). Diet, gut microbiota, and vitamins D + A in multiple sclerosis. Neurotherapeutics.

[28] Zumaraga M.P., Desmarchelier C., Gleize B., Nowicki M., Ould-Ali D., Landrier J.F., Borel P. (2024). Identification of genetic polymorphisms associated with interindividual variability of vitamin A concentration in adipose tissue of healthy male adults. J. Nutr..

[29] Maalmi H., Walter V., Jansen L., Owen R.W., Ulrich A., Schöttker B., Chang-Claude J., Hoffmeister M., Brenner H. (2018). Dose-response relationship between serum retinol levels and survival in patients with colorectal cancer: Results from the DACHS study. Nutrients.

[30] Adler A.J., Wiley G.B., Gaffney P.M. (2013). Infinium assay for large-scale SNP genotyping applications. J. Vis. Exp..

[31] Phan L., Jin Y., Zhang H., Qiang W., Shekhtman E., Shao D., Revoe D., Villamarin R., Ivanchenko E., Kimura M. (2020). ALFA: Allele Frequency Aggregator. https://www.ncbi.nlm.nih.gov/snp/docs/gsr/alfa/.

[32] Tian H.Q., Chen X.Y., Lu Y., Lu W.M., Wang M.L., Zhao H.L., Lu M.P., Zhou H., Chen R.-X., Zhang Z.-D. (2015). Association of VDR and CYP2R1 polymorphisms with mite-sensitized persistent allergic rhinitis in a Chinese population. PLoS ONE.

[33] Koplin J.J., Suaini N.H., Vuillermin P., Ellis J.A., Panjari M., Ponsonby A.L., Peters R.L., Matheson M.C., Martino D., Dang T. (2016). Polymorphisms affecting vitamin D-binding protein modify the relationship between serum vitamin D (25[OH]D_3_) and food allergy. J. Allergy Clin. Immunol..

[34] de Luis D., Izaola O., Primo D., López Gómez J.J., Pacheco D. (2024). Role of rs2282679 in 25-hydroxyvitamin D levels and insulin resistance after a sleeve gastrectomy. Nutr. Diabetes.

[35] Poon A.H., Laprise C., Lemire M., Montpetit A., Sinnett D., Schurr E., Hudson T.J. (2004). Association of vitamin D receptor genetic variants with susceptibility to asthma and atopy. Am. J. Respir. Crit. Care Med..

[36] Baba S.M., Shafi T., Rasool R., Hameed A., Shafi S., Ahmad S.F. (2024). Molecular investigation of vitamin D receptor (VDR) genetic variants and their impact on VDR mRNA and serum vitamin D levels in allergic rhinitis in an Indian population: A case-control study. Int. J. Immunogenet..

[37] Divanoglou N., Komninou D., Stea E.A., Argiriou A., Papatzikas G., Tsakalof A., Pazaitou-Panayiotou K., Georgakis M.K., Petridou E. (2021). Association of vitamin D receptor gene polymorphisms with serum vitamin D levels in a Greek rural population (Velestino study). Lifestyle Genom..

[38] Usategui-Martín R., De Luis-Román D.A., Fernández-Gómez J.M., Ruiz-Mambrilla M., Pérez-Castrillón J.L. (2022). Vitamin D receptor (VDR) gene polymorphisms modify the response to vitamin D supplementation: A systematic review and meta-analysis. Nutrients.

[39] Schulster M.L., Goldfarb D.S. (2020). Vitamin D and kidney stones. Urology.

[40] Ferrucci L., Perry J.R., Matteini A., Perola M., Tanaka T., Silander K., Rice N., Melzer D., Murray A., Cluett C. (2009). Common variation in the beta-carotene 15,15′-monooxygenase 1 gene affects circulating levels of carotenoids: A genome-wide association study. Am. J. Hum. Genet..

[41] Talaei M., Hughes D.A., Mahmoud O., Emmett P.M., Granell R., Guerra S., Shaheen S.O. (2021). Dietary intake of vitamin A, lung function and incident asthma in childhood. Eur. Respir. J..

[42] Su J., Li T., Pan H. (2022). Association of vitamin A supplementation with immune-related allergic diseases: A meta-analysis. Front. Nutr..

[43] Milner J.D., Stein D.M., McCarter R., Moon R.Y. (2004). Early infant multivitamin supplementation is associated with increased risk for food allergy and asthma. Pediatrics.

[44] Bäck O., Blomquist H.K., Hernell O., Stenberg B. (2009). Does vitamin D intake during infancy promote the development of atopic allergy?. Acta Derm.-Venereol..

